# RNA-based therapeutics: an overview and prospectus

**DOI:** 10.1038/s41419-022-05075-2

**Published:** 2022-07-23

**Authors:** Yiran Zhu, Liyuan Zhu, Xian Wang, Hongchuan Jin

**Affiliations:** 1 Laboratory of Cancer Biology, Key Lab of Biotherapy in Zhejiang Province, Cancer Center of Zhejiang University, Sir Run Run Shaw Hospital, School of Medicine, Zhejiang University, Hangzhou, Zhejiang China; 2 Department of Medical Oncology, Sir Run Run Shaw Hospital, School of Medicine, Zhejiang University, Hangzhou, Zhejiang China

**Keywords:** Antisense oligonucleotide therapy, Drug delivery

## Abstract

The growing understanding of RNA functions and their crucial roles in diseases promotes the application of various RNAs to selectively function on hitherto “undruggable” proteins, transcripts and genes, thus potentially broadening the therapeutic targets. Several RNA-based medications have been approved for clinical use, while others are still under investigation or preclinical trials. Various techniques have been explored to promote RNA intracellular trafficking and metabolic stability, despite significant challenges in developing RNA-based therapeutics. In this review, the mechanisms of action, challenges, solutions, and clinical application of RNA-based therapeutics have been comprehensively summarized.

## Facts

- Small molecules and antibody drugs target only 0.05% of the human genome, and most disease targets lack defining active sites for small-molecule binding.
- Abundant RNAs selectively act on proteins, transcripts and genes, which broaden the range of druggable targets. A defined sequence of RNA drugs makes the design of RNA therapeutics much easier.
- Multiple RNA drugs are approved and a dozen more are in phase III trials to treat rare and common diseases.

## Open questions

- How do RNA molecules function in treating diseases?
- The challenges and solutions to the discovery and development of RNA-based drugs effectively.

## Introduction

**Fig. 1:**
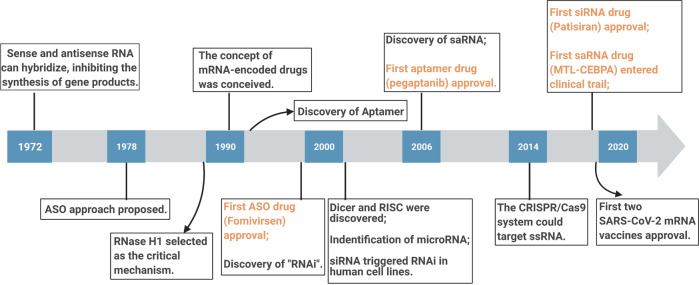
An overview of major developments in the RNA-targeting field. A timeline of RNA-based technological advances, drug approvals and other important events are highlighted. Rapid development in the RNA-targeting field has been applied to treat rare and common diseases. ASOs antisense oligonucleotides, RNAi RNA interference, RISC RNA-induced silencing complex, siRNA small interfering RNA, saRNA small activating RNAs, ssRNA single-stranded RNA.

The antisense oligonucleotides (ASOs) inhibiting protein synthesis in the early 1980s promoted the rapid advances of RNA-based therapeutics [1]. In the 2000s, the proposal of RNAi and the use of siRNA silencing the human gene led to increased funding for RNA therapeutics [2]. Other RNA molecule regulators and related mechanisms also have been well characterized. So far, there are several approved RNA-based medications and many in phase III studies [3] (Fig. 1).

Compared to conventional protein-targeted and DNA-based medicines, RNA-based therapeutics are prospective due to their distinct physicochemical and physiological properties [4]. RNAs function in three essential biological macromolecules: DNAs, RNAs, and proteins. RNA molecules such as ASOs, small interfering RNA (siRNAs), and microRNAs (miRNAs) can directly target mRNAs and noncoding RNAs (ncRNAs) through Watson–Crick base-pairing [4]. Therefore, RNA can theoretically target any interest gene by selecting the correct nucleotide sequence on the target RNA. By contrast, only 0.05% of the human genome has been drugged by the currently approved protein-targeted therapeutics (small-molecule chemicals and antibodies) since most DNA sequences of the human genome are transcribed into noncoding transcripts [5]. Besides, around 85% of proteins lack specific clefts and pockets for small molecules binding [6]. In addition, in vitro transcribed (IVT) mRNA can be applied for protein replacement treatment or immunization after entering the cytoplasm [7]. This process would not cause irreversible genome changes and induce genetic risks like DNA-based therapeutics [8]. Moreover, clustered regularly interspaced short palindromic repeat (CRISPR)-based genome editing can directly modify target RNA sequences to treat specific disorders [9]. RNA aptamers can also block protein activity, similar to small-molecule inhibitors and antibodies [10]. Therefore, RNA-based therapies can broaden the range of druggable targets and are regarded as the most attractive therapeutic target.

This review provides an overview of significant developments in RNA-based therapeutics. The classification of RNA-based therapies and their modes of action have been outlined. This review also covers critical challenges in applying these RNA therapies and possible solutions. Finally, the current preclinical and clinical trials of RNA therapeutics have also been summarized.

## Types of RNA-based therapeutics and modes of action

### Antisense oligonucleotides (ASOs)

**Fig. 2:**
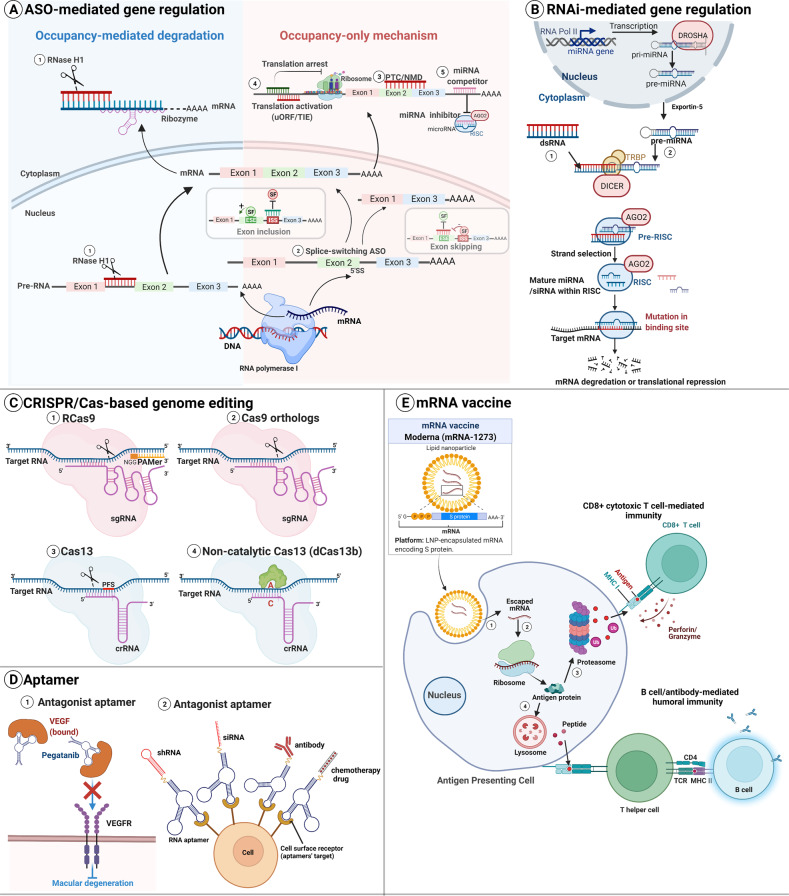
Types of RNA-based therapeutics and modes of action. **A** Antisense oligonucleotides (ASOs) can modulate the target gene expression through two mechanisms. (①) In the occupancy-mediated degradation way, ASOs trigger the target mRNA cleavage by RNase H1 or ribozymes. The Occupancy-only mechanisms do not directly degrade target RNA. Instead, it regulates the gene expression in several ways: (②) alter RNA splicing using splice-switching ASOs to induce exon skipping or exon inclusion; (③) lead to nonsense-mediated mRNA decay (NMD); (④) inhibit or activate translation; (⑤) block the microRNAs binding to target mRNA. **B** RNA interference (RNAi). Long double-stranded RNA (dsRNA) and precursor microRNA (pre-miRNA) are processed by Dicer into short interfering RNA (siRNA). The antisense strand (indicated as a blue strand) of siRNA is loaded into the RNA-induced silencing complex (RISC) for RNA targeting, degradation or translation repression. **C** CRISPR/Cas-based RNA editing system includes two Cas nuclease categories, Cas9 and Cas13. A guide RNA(gRNA) binds to Cas9 to cleave ssRNA with (①) or without (②) the help of a protospacer-adjacent motif (PAM). (③) A single CRISPR RNA (crRNA) guides Cas13 to target specific RNA having a protospacer flanking sequence (PFS). (④) In addition to knockdown target RNA, a catalytically deactivated Cas13b (dCas9b) facilitates the A-to-I editing with ADAR. **D** RNA aptamers function as agonizts or delivery agents. (①) As an antagonist aptamer, Pegaptanib interacts explicitly with vascular endothelial growth factor (VEGF) to inhibit the interaction of VEGF with its receptors, thus treating macular degeneration. (②) Cell type-specific RNA aptamers deliver agents (miRNA, siRNA, shRNA, antibody and chemotherapy drugs) by linking to or conjugating. **E** mRNA vaccine. (①) The mRNA vaccine against SARS-CoV-2 (mRNA-1273) is delivered into antigen-presenting cells by lipid nanoparticle (LNP). (②)The mRNA encoding SARS-CoV-2 spike protein is released into the cytoplasm and translated to antigen protein by the ribosome. (③) Some antigen proteins are degraded into small peptides by the proteasome and presented to the surface of CD8+ T cells by major histocompatibility complex I (MHCI). The CD8+ cytotoxic T-cell-mediated immunity kills infected cells by secreting perforin or granzyme. (④) Other antigen proteins are degraded in the lysosome and displayed on the surface of T helper cells by MHC II. The B-cell/antibody-mediated humoral immunity uses antibodies to neutralize pathogens.

The ASOs are short single-stranded (ss) oligonucleotides (12–24 nucleotides (nt)) complementary to the specific RNA through Watson–Crick base-pairing. They can alter RNA and reduce, restore, or modify protein expression [11]. ASOs are divided into occupancy-mediated degradation and occupancy-only models based on different post-hybridization mechanisms [11] (Fig. 2A).

In the occupancy-mediated degradation, ASOs bind to and cleave the target RNA at the ASO binding sites through endogenous enzymes, enhancing the downregulation of target transcripts (Fig. 2A①). This mechanism is also known as enzymatic RNA degradation since it depends on specific enzymes [11]. RNase H1-mediated degradation is the most well-defined mechanism, where RNase H1 acts as a highly selective endonuclease to cleave the RNA-DNA heteroduplex explicitly. RNase H1 easily targets both the cytoplasmic and nuclear transcripts due to its ubiquitous distribution [12]. Other enzymes, such as ribozymes, can also mediate occupancy-mediated degradation [13]. The ribozyme cleaves target RNAs through hammerhead or hairpin structures [14]. And its substrate recognition domain can be modified to promote cis or trans-site-specific cleavage further.

In the occupancy-only mechanisms, ASOs control the down-/ up-regulation of target transcripts by binding to them without the help of specific enzymes. Sometimes this mechanism is also called the steric block mechanism. Altering RNA splicing is the most widely used strategy [15]. Splice-switching ASOs can change the splicing pattern by targeting splicing regulatory cis-elements [16]. Cis-acting elements activate or inhibit adjacent splice sites through recruiting trans-splicing factors. They consist of splicing enhancers and silencers. When ASOs base-pairing with a splicing enhancer sequence, the stimulatory splicing factor can not bind to its cognate enhancer-binding site, inhibiting splicing and causing exon skipping. Conversely, ASOs target a splicing silencer sequence element that blocks the binding of the inhibitory splicing factor. As a result, the silencer element negatively regulates splicing activation at the splice site, resulting in exon inclusion (Fig. 2A②). Eteplirsen modulates exon skipping [17], and nusinersen induces exon inclusion [18]. In the application part, we discuss these two splice-modulating ASO drugs, eteplirsen and nusinersen. Occupancy-only ASOs can also activate endogenous cellular surveillance programs that remove abnormal mRNAs. The targets can be degraded via nonsense-mediated mRNA decay (NMD) when ASOs act on pre-mRNAs to create mRNAs with premature termination codons (PTCs) [19] (Fig. 2A③). Moreover, ASOs can inhibit or activate translation. On the one hand, ASOs can downregulate the target RNA via translational arrest [20], 5’cap inhibition [21], or polyadenylation changes [22]. On the other hand, ASOs can also bind to inhibitory elements, such as upstream open reading frames (uORFs) [23] or other translation inhibitory components (TIEs) [24], thus upregulating target RNAs (Fig. 2A④). Furthermore, ASOs can inhibit miRNA-mediated downregulation by directly binding to miRNAs (miRNA inhibitors or antagomirs) [25] or combining with mRNAs at miRNA-binding sites to inhibit miRNA interaction (miRNA competitors or block-mir) [26] (Fig. 2A⑤). Occupancy-only ASOs allow more nucleotide modifications than RNase H1-dependent ASOs, thus improving ASO drug properties. Notably, occupancy-only ASOs should not form RNA-DNA duplex to avoid forming RNase H1or Ago2 substrates and unnecessary cleavage of target RNA.

### RNA interference (RNAi)

RNAi is an endogenous cellular process inducing double-stranded (ds) RNAs -triggered degradation of particular RNA targets, which provide an intrinsic defensive mechanism against invading viruses and transposable elements [2, 27, 28]. siRNAs are short dsRNAs (20-24 nt) with distinct structures containing 5’-phosphate/3’-hydroxyl endings and two 3’-overhang ribonucleotides on each duplex strand [29, 30]. siRNAs can induce RNAi in mammalian cells. Therefore, researchers can use such simple gene silencing tools to investigate gene function and advance disease therapy [31]. Mechanistically, the endoribonuclease Dicer cuts dsRNAs and isolates the guide and passenger strands within the RNA-induced silencing complex (RISC). The argonaute2 (AGO2) protein degrades the passenger siRNA strand, whereas the guide siRNA strand directly binds to the target RNA, causing AGO2-mediated cleavage [32] (Fig. 2B). Besides degrading cytoplasmic RNAs, siRNAs can also trigger chromatin remodeling and histone modifications in the nucleus when they bind to the promoter regions, resulting in transcriptional silence [31, 33].

miRNAs are endogenous single-stranded small ncRNAs influencing gene expression via RNAi [34]. The biogenesis of miRNAs follows a systematic process. First, miRNAs are produced from lengthy primary precursor miRNAs (pri-miRNAs), cleaved in the nucleus by RNase III–family nuclease Drosha. The free pre-miRNAs are then transported to the cytoplasm, where their loop regions are cleaved by Dicer to produce mature miRNA. The mature miRNA duplex (comprising two strands) is loaded into the pre-RISC for strand selection. One of the two miRNA strands is selectively loaded into the miRNA-induced silencing complex (miRISC), whereas the other strand is ejected from the complex and is subject to degradation. Both strands may be loaded into RISC at similar frequencies, while different strand usage depending on biological contexts. The strand from the 5′ end of the stem-loop and the 3′ strand are named “5p” and “3p”, respectively [35, 36]. Finally, miRNAs cause mRNA translational repression or degradation in the miRNA-induced silencing complex (miRISC) by base-pairing with specific RNA sequences (often in the 3′ untranslated region (UTR)) [34] (Fig. 2B). Interestingly, emerging evidence has revealed that miRNAs can upregulate targeted genes through increasing mRNA stability or/and translation [37]. For example, miR-346 targets the *amyloid-β peptide precursor protein (APP)* mRNA 5′-UTR to upregulate APP translation and amyloid-β production [38]. miR-466l elevates *IL-10* mRNA stability and IL-10 protein expression through binding to IL-10 AU-rich elements [39]. miRNAs have crucial roles in regulating gene expression and human diseases. miRNA mimics have been currently in preclinical development as putative therapeutic agents. Chemically modified, completely base-paired siRNAs with the identical guide strand sequence as an endogenous miRNA are widely used as miRNA mimics [40].

Unlike RNAi, RNA activation (RNAa) is a process where dsRNA triggers gene production by targeting promoter sequences [41]. Small activating RNAs (saRNAs) are synthesized using homologous sequences close or within gene promoters, which can trigger RNAa. Similar to miRNA-like target recognition, saRNAs actions depend on the AGO2 protein. In the nucleus, AGO2–saRNA uses the “seed” region to basepair with sequences inside the chromatin-bound RNA transcripts or complementary DNA [41–43]. Besides saRNA and AGO2, recent research found that RNA-induced transcriptional activation (RITA) complex also contains RHA and CTR9 [44]. saRNA can alleviate the downregulation of silent tumor suppressor genes (like p21) or other typical dysregulated genes (like E-cadherin) and thus may promote the development of dsRNA-based therapeutics for cancer and other disorders [45].

### CRISPR-based genome editing

The prokaryote-derived CRISPR-associated protein (Cas) systems have been widely used in mammalian cells and organisms to precisely edit genome sequence, resulting in irreversible knockout or knockin of a target gene [46]. Mechanistically, this system relies on a designed guide RNA (gRNA) and an RNA-guided Cas nuclease. The gRNA forms the Cas-gRNA ribonucleoprotein complex by binding to Cas. The complex recognizes a protospacer-adjacent motif (PAM) element and a 20-nucleotide sequence in the target sequence. The Cas nuclease then cleaves the dsDNA or an ssRNA at the specific site for efficient genome editing [47]. Initial successes have enhanced the development of new methods for targeting and manipulating nucleic acids, such as Cas9 and Cas13 orthologues-derived methods [9]. The Cas9 system can target both dsDNA and ssRNA. The Cas9 target RNA of *Streptococcus pyogenes* (RCas9) requires a matching gRNA and complementary PAM-presenting oligonucleotide (PAMmer) [48] (Fig. 2C①). Cas9 orthologs (Cas9 of *Campylobacter jejuni* and *Staphylococcus aureus*) can cleave ssRNA without PAM [49] (Fig. 2C②). Cas13-mediated systems only target RNA, where a CRISPR RNA (crRNA) guides Cas13 to cleave specific RNA. Cas13a, Cas13b and Cas13d have been verified to interfere with and silence target RNA in mammal cells in vitro. CasRx (RfxCas13d), a subtype of Cas13d, showed the most potent RNA knockdown efficiency in HEK293T cells [50]. A protospacer flanking sequence (PFS) may be required to cut the target and non-target RNA molecules via two conserved Higher Eukaryotes and Prokaryotes Nucleotide-binding (HEPN) domains [51] (Fig. 2C③). Cas13d is a new PFS-independent Cas, and a non-catalytical variant of Cas13b that lacks endonuclease activity can induce the A-to-I base switch by fusing with the ADAR2 deaminase domain (ADAR2_DD_) [52] (Fig. 2C④).

### Aptamer

Aptamers are single-stranded oligonucleotides with well-defined three-dimensional structures that specifically bind to and inhibit proteins [10]. They are also known as chemical antibodies due to their synthetic origins and similar action modes to antibodies. Systematic evolution of ligands through exponential enrichment (SELEX) is used for aptamer selection [53]. Aptamer-based therapeutics include: (1) Antagonist aptamers disrupt the interaction between disease-associated targets, such as protein-protein or receptor-ligand interactions. All the current aptamers in clinical trials use this strategy [54] (Fig. 2D①). (2) Cell type-specific aptamers serve as carriers to deliver other therapeutic agents to the target cells or tissues [55] (Fig. 2D②).

### mRNAs and mRNA vaccine

The concept of mRNA-encoded drugs was discovered in the 1990s when direct injection of IVT mRNA into the mouse skeletal muscle showed encoded protein expression [56]. Preclinical research on IVT mRNA promotes the clinical development of mRNA-based vaccination against cancer and infectious disease [7]. Mechanistically, injected mRNA vaccines are delivered into the cytoplasm of the host cell (typically antigen-presenting cells (APCs)) and are translated into the targeted antigens. Subsequently, the major histocompatibility complexes (MHCs) present the expressed antigens to the surface of APCs to activate B cell/antibody-mediated humoral immunity and CD4+ T/CD8+ cytotoxic T-cell-mediated immunity [57]. Besides, injected mRNA encoding immunostimulants (cytokines, chemokines, etc.) can promote APC maturation and activation, thus inducing a T-cell-mediated response and improving the immune tumor microenvironment [58] (Fig. 2E).

## Overcoming challenges in the development of RNA therapeutics

The efficiency of RNA delivery into the cytoplasm through overcoming the extracellular and intracellular barriers remains critical for successful RNA therapy. Firstly, large, hydrophilic, negatively-charged properties prevent RNA from passively diffusing across the lipid bilayers. Beyond the physical barrier, RNA drugs must evade serum nucleases and scavenge macrophages within the reticuloendothelial system. Moreover, RNA drugs must pass through the extracellular matrix and across the cell membrane through receptor-mediated endocytosis. Escaping from the endosome and releasing RNAs into the cytoplasm in a non-toxic manner is a critical technical problem [59]. Therefore, various chemical modifications [60, 61] and the engineering of delivery formulations [62, 63] have been explored to solve challenges related to pharmacodynamics and pharmacokinetics.

## Chemical modifications

### Chemical modifications of ASOs and siRNAs

**Fig. 3:**
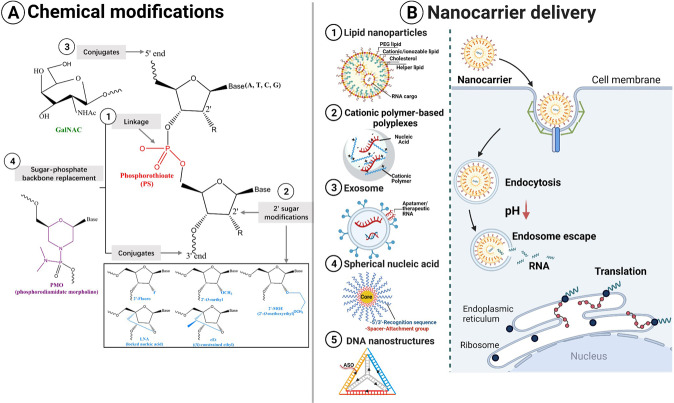
Overcoming challenges in the development of RNA therapeutics. **A** Common chemical modifications. RNA-based drugs often have various chemical modifications, including 5′-and 3′-end conjugates, 2′-sugar substitution and internucleoside linkage modifications. **B** Nanocarriers delivery strategies. Five representative nanocarriers are shown: (①) Lipid nanoparticles encapsulating nucleic acids. (②) Cationic polymers electrostatically bind to negatively-charged nucleic acids to form polyplexes. (③) Engineered exosomes with aptamers or therapeutic RNAs on the outer surface. (④) Spherical nucleic acid nanoparticle consisting of an inorganic core coated in densely packed oligonucleotides attached by chemical linkages. (⑤) Self-assembled DNA cage tetrahedron nanostructure. Oligonucleotide drugs can be incorporated into the design of the DNA cage itself. Additional targeting ligands and polyethylene glycol (PEG) can be further conjugated to the nanostructure. These nanocarriers can deliver RNA molecules through binding to the cell membrane, endocytosis, endosome escape and RNAs are released in the cytoplasm and translation to proteins or incorporated into corresponding ribonucleoprotein complexes to silence target transcripts.

Chemical modification of the phosphate backbone, ribose ring, and 3′- and 5′-terminals can improve substrate specificity, nuclease resistance and delivery. Besides, it reduces toxicity and immunogenicity [64]. The modification of phosphorothioate (PS) backbones (Fig. 3A①) was the first and most commonly used chemical modification in ASOs. In this modification, one of the non-bridging oxygen atoms in the inter-nucleotide phosphate group is replaced with sulfur [65]. The PS backbone modification can facilitate cellular uptake and bioavailability in vivo via increased hydrophobicity, resistance to phosphodiesterases and avidly binding serum proteins [65]. The modifications of 2′ sugar at the ribose, including 2′-fluoro (F), 2′-methoxyethyl (MOE), 2′-O-methyl (O-Me) or 2′,4′-bicyclics with O-methylene bridge or locked nucleic acid (LNA), can improve the binding affinity and increase base-pairing melting temperature [66, 67] (Fig. 3A②). The 2′-F and 2′-O-Me modifications imitate the biophysical features of 2′-OH and can stabilize siRNAs against RNases while also preventing siRNAs activating innate immune receptors (TLR, MDA-5, and RIG-I). As a result, all therapeutic siRNAs in clinical trials have 2′-F or 2′-O-Me modifications [68]. LNA and its methylated derivative (“constrained ethyl” (cEt)) are widely used in ASOs, including gapmers, splice-switching ASOs, siLNA and antigene ASOs. These chimeric LNA or DNA oligonucleotides are more stable than isosequential PSs and 2’-O-Me gapmers [69]. Many 5′- or 3′-RNA conjugates (folate and *N*-acetylgalactosamine (GalNAc)) can also improve delivery. GalNAc is a high-affinity ligand for the hepatocyte-specific asialoglycoprotein receptor (ASGPR), thereby significantly enhancing ASO and siRNA delivery to the liver [70] (Fig. 3A③). Phosphorodiamidate morpholino oligonucleotide (PMO) [71] and peptide nucleic acid (PNA) [72] are more complex modifications that entirely alter the linking moieties while maintaining nucleobases for pairing (Fig. 3A④).

### Chemical modifications of mRNA, CRISPR-Cas guide RNAs, and aptamer

IVT mRNA is an ssRNA containing a 5′ cap structure, an ORF, flanking 5′ and 3′ UTRs and a 3′ poly (A) tail [73]. The optimization of mRNA vaccines often starts from these five structures through sequence optimization, nucleoside modification or sequence substitution of UTRs to enhance RNAs’ translational ability [74]. Various chemical modification nucleosides, including pseudouridine (Ψ), N1-methylpseudouridine (m1Ψ), 5-methylcytidine (m5C), 5-hydroxymethylcytosine (5hmC), 5-methyluridine (m5U) and 2-thiouridine (s2U) have been introduced into mRNA to minimize the innate immune response triggered by IVT mRNAs [75, 76].

Despite their robustness and simplicity, therapeutic use of CRISPR systems has many obstacles, including effective delivery, detectable off-target effects, potential immunogenicity, etc. Chemical modification patterns used in ASOs and siRNAs can be applied to gRNA and Cas9 mRNA [77]. For instance, chemical modifications containing 2′- O-M -3′PS (MS), 2′-O-M, or 2′-OM- 3′thioPACE (MSP) can be integrated into single-gRNAs (sgRNAs) at three terminal nucleotides on both the 5′ and 3′ ends to improve genome editing efficiency in human primary T cells and CD34+ hematopoietic stem and progenitor cells [78]. The ribose-phosphate backbone of gRNAs with 2′-OM-3′-phosphonoacetate (MP) modification can significantly minimize off-target cleavage while maintaining strong on-target performance [79]. The 5′-H group modification of sgRNAs can be highly active and evade innate immune responses [80]. Incorporating bridging nucleic acids (2′,4′-BNANC[N-Me]) and LNA at particular sites in crRNA can significantly reduce off-target DNA cleavage by Cas9 [81]. The chemical modification of IVT mRNA can be used on Cas9 mRNA.

Like ASOs and siRNAs, versatile chemical modifications and conjugations can enhance the pharmacokinetic features of RNA aptamers [82]. Most aptamers in the clinical trials are chemically modified using 5′-end polyethylene glycol(PEG)ylation (for resisting renal clearance) [83], 3′end-capping strategy with inverted thymidine [84], and 2′-substitutions on the sugar ring (for preventing nuclease degradation). PS linkage modification can also optimize the properties of aptamers by improving target binding affinity [85].

## Delivery formulation with nanocarriers

Developing an effective carrier to protect the carried RNA from the harmful physiological environment is necessary since RNAs have substantial negative charges and chemical modifications. Advances in nanotechnology and materials science offer advantages and potential solutions to the challenges of oligonucleotide drug delivery, especially for the requirements of intracellular delivery across biological barriers and membranes. Key benefits of nanoparticle drug delivery systems include custom optimization of nanoparticle biophysics (e.g., size, shape, and chemical/material composition) and biological properties (e.g., targeting ligand functionalization), allowing for a high degree of customization delivery platform. Here we introduced two dominant delivery approaches—lipid nanoparticles and cationic polymer-based polyplexes, and three emerging novel nanocarriers. We summarize the representation of RNA therapeutics delivery as shown in Table 1.

**Table 1:** Delivery method for RNA-based therapeutics.

Delivery method	Composition	Payload	Example	Advantage	Limitation	References
Lipid nanoparticles	DLin-MC3-DMA (MC3)	siRNA	siRNA drug (Patisiran)	Increased half life; protection from nucleases acids	Elevated risk of immunotoxicity and immunogenicity	[86–89, 93–95, 147, 148]
	ALC-0315	mRNA, gRNA	SARS-CoV-2 mRNA vaccine (BNT162b2)	Facilitate the endosomal escape of RNA molecules;increase in protein expression or immune responses in mice compared to MC3		
	SM-102	donor RNA	SARS-CoV-2 mRNA vaccine (mRNA-1273)			
Cationic polymer-based polyplexes	Polyethyleneimine (PEI)	mRNA, siRNA, miRNA, gRNA	SiG12D LODER; CRM197-PEG-PEI-based complexes	High charge density and pH buffer capacity	Potential toxicity and plasma instability	[96–98, 136]
	Chitosan	mRNA, siRNA, (DNA)	siRNA loaded chitosan lactate nanoparticles (CL-TAT-HA)	Low toxicity, biodegradability, biocompatibility, and permeability-enhancing	Low transfection efficiency	[99]
	Cyclodextrin polymer (CDP)	siRNA	CALAA-01			[100, 101]
	Poly(β-amino esters) (PBAEs)	mRNA, gRNA, donor RNA	HPV16 E7-targeting CRISPR/short hairpin RNA (shRNA)	Low toxicity	Low charge density	[102]
Exosomes	Derived sources: mesenchymal stem cells	miRNA, siRNA, mRNA, other ncRNAs	miR-124; *KRAS G12D* siRNA	With minimal immune clearance and adverse effects		[105, 106]
Spherical nucleic acids	AuNP, quantum dots (QDs), SiO2, Ag, Fe3O4,	mRNA, siRNA, gRNA, donor RNA	NU-0129 (Bcl2Like12 (Bcl2L12) siRNA)	Show rapid cellular uptake kinetics and intracellular transport; induce a negligible immune response.		[107, 109, 110]
			AST-005 (inhibiting TNF-α mRNA via ASOs); XCUR17 (interleukin-17 receptor-α through ASOs)			
DNA nanostructures		ASOs, siRNAs, aptamers, CRISPR-Cas9	AS1411 aptamers	The object’s size, shape and plasticity can be fine-tuned	Enzymatic hydrolysis, low cellular uptake, immune cell recognition and degradation, and unclear biodistribution profiles	[111–114, 117]

### Lipid nanoparticles (LNPs)

LNPs are the most widely used carriers to deliver oligonucleotide drugs [86, 87]. LNPs consist of ionizable cationic lipids, cholesterol, phospholipids and PEG-lipids (Fig. 3B①). Ionizable cationic lipids are the core components. Cationic lipids form “lipoplexes” by electrostatically binding to negatively-charged nucleic acids, which have been widely used in vitro for nucleic acid transfections (e.g., Lipofectamine™ RNAiMAX transfection reagents). Helper lipids, phospholipids and cholesterol promote formulation stability and delivery efficiency [88]. PEG-lipid can control particle size, prevent particle aggregation, and lengthen in vivo circulation lifetimes [89]. Other reviews have comprehensively discussed the advanced formulation of LNPs’ optimization characteristics and production methods [90].

Using the LNP-mediated siRNA delivery as an example [91] (Fig. 3B). The acid dissociation constant (pKa) determines the nanoparticles’ ionization behavior and surface charge, thereby influencing the delivery process. Firstly, positively charged LNPs prevent anionic RNAs from nucleases by coating RNAs and help RNAs across the cell membrane through receptor-mediated endocytosis. After entering the cells, the charges on the nanoparticle increase as the pH decrease (from 7 to 5.5) during endosomal maturation. Nanoparticles with a pKa in this range are protonated and create a buffering capacity. The buffering capacity of nanoparticles and/or membrane destabilization causes osmotic swelling and endosome breaking. The charges on nanoparticles decrease in the cytosol and weaken the binding to siRNAs [92]. The siRNAs then escape from endosomes into the cytosol, the critical rate-limiting step for its delivery. As a result, siRNAs cleave target RNAs by associating with the RISC. Other RNA entities may be translated to proteins or translocated in the nucleus. Therefore, optimizing LNPs’ pKa dramatically increases the delivery efficiency of RNA drugs. Besides, LNP-loaded RNA entities may be safely and effectively delivered to specific cells, organs, and tissues using the emerging advanced technologies. The branched-tail LNP can sufficiently co-deliver three distinct mRNAs in vivo and are not immunogenic or toxic to the liver [93]. The engineered-ionizable LNP has been developed for selective delivery of RNA into various liver cells [94]. The selective organ targeting (SORT) technique could specifically target the liver and extrahepatic tissues (lung and spleen) by adding a SORT molecule into LNP. This technique enables mRNA delivery and CRISPR-Cas gene editing in specific tissues [95]. Therefore, LNP-based gene therapies can treat hepatic diseases and other rare diseases.

### Cationic polymer-based polyplexes

Polyplexes are standard formulations used for nucleic acid delivery. They are spontaneously formed by electrostatic interactions between cationic polymers and negatively-charged nucleic acids (Fig. 3B②). The polyethyleneimine (PEI) polymer family is the most widely studied polymeric material for nucleic acid delivery. They consist of linear or branched polycations that can form nanoscale complexes with miRNA or siRNA, thus leading to RNA protection and cellular delivery. A commercially available linear PEI derivative, jetPEI™, is widely used for DNA, siRNA and mRNA transfection. Besides, transforming RNA vaccines from PEI functionalized graphene oxide hydrogel in situ has been used for cancer immunotherapy effectively [96]. Targeted CRM197-PEG-PEI-based complexes for siRNA delivery in vivo show therapeutic effects by knockdown of growth factor pleiotrophin in glioblastoma [97]. However, PEI is relatively cytotoxic and not degradable [98]. Chitosan is a biopolymer found in the exoskeleton of crustaceans. Chitosan and its variants have been developed for DNA and siRNA delivery due to their biodegradability, biocompatibility and permeability-enhancing properties. However, the low transfection efficiency limits their clinical application [99]. Chitosan grafted PEI has been synthesized with increased transfection efficiency and lower toxicity [98]. Cyclodextrin polymer (CDP)-based nanoparticles were the first targeted nanoparticulate siRNA delivery system, which enters clinical trials for cancer. The self-assembling, CDP-based nanoparticle (denoted as CALAA-01) targets the *ribonucleotide reductase M2* mRNA in patients with solid cancers [100, 101]. Other polymers like poly(β-amino esters) (PBAEs) [102] and polyaspartamides [103] are also used in nucleic acid delivery. However, there have been no polyplexes nanoparticles for RNA delivery approved. Therefore, improved strategies are required for producing scalable cationic polymer-based polyplexes with high target specificity and less toxic or immunogenic.

### Emerging novel approaches-exosomes, spherical nucleic acids (SNAs), and DNA nanostructures

Exosomes are lipid membrane-enclosed vesicles with a diameter ranging from 40 to 160 nm (Fig. 3B③). They are found in all body fluids and are secreted by most cells. Exosomes are crucial intercellular communication mediators influencing many cell biology processes [104]. They can transport bioactive constituents and overcome biological barriers (blood–brain barrier). As a result, exosomes have attracted much attention as potential delivery vehicles for therapeutic agents. Engineered exosomes can deliver different RNA species with minimal immune clearance and adverse effects [105, 106].

SNAs are densely packed, radially oriented nucleic acids in the outer layer and covalently bond to an inorganic nanoparticle core. Gold nanoparticles (10–15 nm) are the most commonly used SNA cores. Alkyl thiol or cyclic disulfide chemical tethering groups are used to attach nucleic acids to the core surface. A recognition sequence (15–25 nt) is complementary to the target sequence, and a spacer (10 nt) connects the nanoparticle surface with the recognition sequence [107] (Fig. 3B④). SNAs can effectively deliver nucleic acid because: (1) they show rapid cellular uptake kinetics and intracellular transport. Their 3D structure can be recognized by class A scavenger receptors. Then, the proximity of SNAs, class A scavenger receptors and lipid-raft microdomains allow SNAs to be rapidly internalized through endocytosis in a lipid-raft-dependent and caveolae-mediated manner [108]. (2) they induce a negligible immune response. The high density of nucleic acids at the surface of SNAs inhibits nucleases-mediated degradation. Therefore, they can enter almost any cell type and various biological barriers (skin, tumor, and blood–brain barrier) without using transfection agents. Delivery of SNAs by exosomes, modification of the oligonucleotide sugar backbone or PEGylation of nanoparticles could further enhance the effectiveness of SNAs [107]. Exicure, Inc. has developed SNA therapeutic platforms with four SNA-loaded drugs currently in clinical trials. Systematic delivery of SNAs carrying Bcl2Like12 (Bcl2L12) siRNA (NU-0129) can act as a brain-penetrant precision therapeutic strategy for intracerebral glioblastoma multiforme treatment [109]. AST-005 can treat psoriatic lesions by inhibiting *TNF-α* mRNA via ASOs. XCUR17 can target interleukin-17 receptor-α through ASOs for treating psoriasis micro plaques. ST-008, TLR9 agonist SNA, combined with immune checkpoint inhibitors, are used for immuno-oncology treatments [110].

DNA-based nanostructures are also widely used as delivery vehicles (Fig. 3B⑤). The self-assembly features of DNA allow it to fold into user-defined shapes through sequence-complementary domain hybridization. As a result, the object’s size can be precisely controlled at the nanometer scale, and the object’s shape and plasticity can be fine-tuned [111]. DNA nanostructures deliver RNA by incorporating ASOs or siRNAs on the structure surface [112]. AS1411 aptamers can also be inserted into DNA pyramids to promote intracellular uptake and nuclease degradation resistance [113]. The yarn-like DNA nanoclew can deliver CRISPR-Cas9 through rolling circle amplification and base-pairing with the Cas9/sgRNA complex [114]. Moreover, cytosine-phosphate-guanine (CpG) oligonucleotides can target tissue macrophages when incorporated into DNA nanotubes by enhancing macrophage immunostimulation [115, 116]. However, the high costs of DNA and the high error rate of self-assembly limit the application of DNA nanostructures in emerging complex structures [117].

## Application

RNA-based therapeutics were firstly adopted for diseases with clear rationales, such as oncology, neurological disorders and infectious diseases. Recent advancements in RNA technologies have led to the approval of eight ASOs, three siRNAs, and two mRNA vaccines for COVID-19 under the Emergency Use Authorization scheme (Table 2). Besides, other medicines are currently undergoing preclinical or clinical trials. Moreover, more advanced RNA-based therapeutics have been developed recently.

**Table 2:** RNA-based drugs with either FDA approval or in late phase 3 clinical trials.

RNA class	Drug	Alternative name	Sponsor	Indication	Target gene	Target organ	Chemical modifications or delivery method	Administration route	Updated states	Comments	References
ASO	Fomivirsen	Vitravene, ISIS 2922	Ionis Pharmaceuticals	Cytomegalovirus Retinitis, HIV Infections	*Immediate early region 2 (IE2)* mRNA	Eye	2′-H	Intravitreal	Completed NCT00002187, NCT00002355	The first FDA-approved ASO medication (1998), withdrawal in Europe and USA in 2002	[118, 119]
	Mipomersen	Kynamro™, ISIS 301012	Kastle and IonisTherapeutics	Hypercholesterolemia, Atherosclerosis, Coronary Artery Disease	*Apolipoprotein B (ApoB)* mRNA	Liver	2′-MOE	Subcutaneous	Completed NCT01598948 NCT00607373 NCT01475825 NCT00770146 NCT00694109 NCT00794664 NCT00706849	The second-generation of ASOs (“Gapmers” ASO)	[120]
	Inotersen	ISIS 420915, Tegsedi, AKCEA-TTR-LRx	Ionis Pharmaceuticals	Hereditary ATTR Amyloidosis (hATTR)	*Hepatic transthyretin (TTR)* mRNA	Liver	2′-MOE	Subcutaneous	Completed NCT01737398		
	Nusinersen	ISIS 396443, Sprinraza, IONIS-SMN Rx, BIIB058	Ionis Pharmaceuticals and Biogen	Spinal Muscular Atrophy (SMA)	*Survival of motor neuron 2(SMN2)* pre-mRNA splicing (exon 7 inclusion)	Central nervous system	2′-MOE, Fully modified	Intrathecal	Completed NCT02292537	Approved in 2016	[18, 121]
	Eteplirsen	AVI-4658, EXONDYS 51^®^	Sarepta Therapeutics	Duchenne Muscular Dystrophy (DMD)	*DMD* pre-mRNA splicing (exon 51 skipping)	Muscule	2′-MOE, PMO	Intravenous	Completed NCT02255552	The third-generation of ASO medications with advanced chemical modifications	[17, 121]
	Golodirsen	SRP-4053,Vyondys 53™	Sarepta Therapeutics	Duchenne Muscular Dystrophy (DMD)	*DMD* pre-mRNA splicing (exon 53 skipping)	Muscle	2′-MOE, PMO	Intravenous	Recruiting NCT02500381		[122]
	Viltolarsen	Viltepso, NS-065, NCNP-01	NS Pharma	Duchenne Muscular Dystrophy (DMD)	*DMD* pre-mRNA splicing (exon 53 skipping)	Muscle	2′-MOE, PMO	Intravenous	Recruiting NCT04768062 NCT04687020 NCT04060199		[123]
	Casimersen	SRP-4045, Amondys 45™	Sarepta Therapeutics, Inc.	Duchenne Muscular Dystrophy (DMD)	*DMD* pre-mRNA splicing (exon 45 skipping)	Muscle	PMO	Intravenous	Recruiting NCT03532542 NCT02500381		[124]
RNAi	Patisiran	ALN-TTR02, ONPATTRO™	Alnylam Pharmaceuticals	hATTR	*TTR* mRNA	Liver	PS, 2′-O-Me, 2′-F (LNP)	Intravenous	NCT03862807, NCT01960348	The first siRNA drug approved by FDA (2018)	[129]
	Givosiran	ALN-AS1, GIVLAARI	Alnylam Pharmaceuticals	Acute Hepatic Porphyria	*Aminolevulinate synthase 1(ALAS1)* mRNA	Liver	PS, 2′-O-Me, 2′-F GalNAc	Subcutaneous	NCT03338816	The second siRNA drug approved by FDA (2019)	[134]
	Lumasiran	ALN-GO1, OXLUMO	Alnylam Pharmaceuticals	Primary Hyperoxaluria Type 1 (PH1)	*Hydroxyacid oxidase 1 (HAO1)* mRNA	Liver	PS, 2′-O-Me, 2′-F GalNAc	Subcutaneous	Phae III active; NCT03905694 NCT03681184 NCT04152200	The third siRNA drug approved by FDA (2020)	[135]
	Inclisiran	ALN-PCSSC, LEQVIO	Alnylam and Novartis Pharmaceuticals	Hypercholesterolemia, Atherosclerotic Cardiovascular disease, Renal impairment	Proprotein convertase subtilisin kexin type 9 (PCSK9) mRNA	Liver	PS, 2′-O-Me, 2′-F GalNAc	Subcutaneous	Phase III completed; NCT03399370 NCT03400800 NCT03397121	Expand siRNA’s clinical portfolio beyond just orphan diseases.Provide sustained reductions in low-density lipoprotein (LDL) cholesterol levels with infrequent dosing (every 6 months).	
	Vutrisiran	ALN-TTRSC02	Alnylam Pharmaceuticals	hATTR	*TTR* mRNA	Liver	PS, 2′-O-Me, 2′-F GalNAc	Subcutaneous	Phae III active; NCT04153149 NCT03759379	May prove to be a more clinically utilize, effective treatment option for hATTR than patisira	
	Fitusiran	ALN-AT3SC	Alnylam Pharmaceuticals and Sanofi Genzyme	Hemophilia A / B	*Antithrombin* mRNA	Blood	PS, 2′-O-Me, 2′-F GalNAc	Subcutaneous	Phase III completed; NCT03974113 NCT03417102		
	Nedosiran	DCR-PHXC	Dicerna Pharmaceuticals	Primary Hyperoxaluria	*Hepatic lactate dehydrogenase* mRNA	Liver	GalNAc	Subcutaneous	Phase III enrolling by invitation; NCT04042402	Compare with Lumasiran,it will have a potentially wider scope, since it is not limited to just PH1 patients.	
	Teprasiran	QPI-1002	Quark Pharmaceuticals	Cardiac surgery	*p53* mRNA	Kidney	2′-O-Me	Intravenous	Phase III completed; NCT02610296	The first systemically administered siRNA drug to enter human clinical trials	
	QPI-1007		Quark Pharmaceuticals	Primary angle-closure glaucoma	*Caspase 2* mRNA	Eye	2′-O-Me	Intravitreal	Phase II/III terminated: NCT02341560		
	Tivanisiran	SYL-1001	Sylentis, S.A.	Dry eye disease	*Transient receptor potential cation channel subfamily V member 1 (TRPV1)* mRNA	Eye	Unmodified	Topical eye drop	Phase III completed; NCT03108664		
Aptamer	Pegaptanib	Macugen^®^	Pfizer	Diabetic Macular Edema	VEGF (165 isoform)	Eye	Pegylated, all PO, 2′-F, and 2′-OMe; G and A methylated	Intravitreal injection	Phase IV Completed; NCT01486238 NCT01486238 NCT00406107 NCT00324116		[146]
mRNA vaccine	BNT162b2	Comirnaty^®^	BioNTech and Pfizer	Coronavirus disease 2019 (COVID-19)	Encodes the SARS-CoV-2 spike protein	Immune system	Nucleoside-modifed, lipid nanoparticle-formulated	Interventional	Completed NCT04816669 NCT04887948 NCT04713553 NCT05030974	The first two FDA-approved SARS-CoV-2 vaccines with >94% effectiveness in phase III clinical trial	[147]
	mRNA-1273		Moderna Therapeutics	COVID-19	Encodes the SARS-CoV-2 spike protein	Immune system	Lipid nanoparticle-formulated	Interventional	Completed NCT05030974		[148]

### Performance of ASOs in clinical trials

Out of the 100 phase I trials performed on ASO-based therapies, a quarter has entered phase II/III trials in the last 5 years for treating rare and common diseases, such as orphan genetic alterations and cancer [11].

Fomivirsen is the first FDA-approved ASO drug for treating cytomegalovirus retinitis (CMV) in patients with AIDS [118]. This PS ASO complementary to human CMV immediate-early mRNA inhibits viral protein synthesis and interrupts viral replication [119]. However, since the introduction of highly active anti-retroviral therapy, cases of CMV have significantly reduced. Therefore, fomivirsen was withdrawn from the market in Europe and USA in 2002. Mipomersen and inotersen are second-generation ASO drugs called “Gapmers” or chimeric ASOs. Mipomersen effectively degrades *apolipoprotein B (ApoB)* mRNA. Likewise, inotersen mediates RNase H1-mediated degradation of *hepatic transthyretin (TTR)* mRNAs to reduce TTR protein synthesis and serum TTR levels [120]. Two splice-modulating ASOs (nusinersen and eteplirsen) were approved in 2016 for treating splicing defects [121]. Nusinersen is the first splicing-correcting ASOs approved for spinal muscular atrophy (SMA) treatment. The 2′-MOE ASO promotes exon 7 inclusion and increases the expression of SMN protein by binding to the intronic splicing inhibitor in intron 7 [18]. Eteplirsen is another splice-modulating oligonucleotide used to treat Duchenne muscular dystrophy (DMD) patients. It hybridizes to a splicing enhancer sequence in exon 51 that causes the spliceosome ignores exon 51 and directly read exon 52 in the frame, thus producing shorter yet semi‐functional dystrophin proteins [17]. Since eteplirsen is suitable for only 13–14% of DMD patients with specific mutations, golodirsen [122], viltolarsen [123], and casimersen [124] have been approved for the treatment of DMD with particular splicing defects. These drugs induce exon 53 or 45 skippings, thereby promoting the expression of dystrophin proteins. Together with eteplirsen, these four drugs are third-generation ASO drugs belonging to PMOs sophisticated chemical modifications. Because of the urgency of the patient’s clinical situation and ASO medications may be modified in a sequence-specific manner, patient-customized oligonucleotide treatment has been developed. Milasen is the first patient-customized ASO for neuronal ceroid lipofuscinosis 7 disease (a fatal neurodegenerative disease). It showed an acceptable side-effect during the therapy [125].

Several clinical trials are being conducted to test the efficacy of ribozymes in the treatment of solid tumors, HIV and other diseases [126]. RPI.4610 (Angiozyme) is a ribozyme that inhibits angiogenesis by targeting the *vascular endothelial growth factor receptor one (VEGFR1)* mRNA. However, the poor efficacy prevents its clinical development [127]. OZ1 is a tat-vpr-specific anti-HIV ribozyme that increases the number of CD4+ lymphocytes when administered to autologous CD34+ hematopoietic progenitor cells. This suggests that cell-mediated delivery of genes is a safe therapeutic approach for HIV patients and is likely to be a conventional treatment for HIV [128]. Although the results obtained in the initial clinical trials were positive, further investigation is required to determine the stability, in vivo activity, tissue-specific delivery, and long-term expression of ribozymes [126].

### RNAi-based therapy

#### Patisiran and GalNAc-conjugated siRNAs

Three siRNA drugs (patisiran, givosiran, and lumasiran) have been approved by FDA to date, while seven siRNA candidates (inclisiran, vutrisiran, fitusiran, cosdosiran, nedosiran, tivanisiran and teprasiran) are undergoing Phase III clinical trials.

Patisiran is the first FDA-approved RNAi-based drug for treating hTTR with polyneuropathy, ushering in a booming new era for RNAi therapeutics [129]. Similar to inotersen [130], the patisiran siRNA (ALN-18328) silences all potential mRNAs with coding region mutations by targeting the 3′ UTR of the TTR gene [129]. Alnylam developed the stabilization chemistry-GalNAc delivery platform to improve the clinical efficacy of siRNA medications. To date, approximately one-third of RNAi drugs in clinical trials are GalNAc-conjugated siRNAs. Revusiran was the first GalNAc–siRNA drug that significantly increased asialoglycoprotein receptors uptake for hepatic delivery [131]. Unfortunately, it was discontinued due to an imbalance in fatalities in the “ENDEAVOUR” phase III clinical trial (NCT02319005) [132]. Despite the failure of revusiran, Alnylam has continued to develop GalNAc–siRNA conjugates for therapeutic usage by strategically positioning chemical modifications within the siRNA that can impart extra stabilization against nuclease activity [133]. Givosiran [134] and Lumasiran [135], the second and third siRNA drugs approved by the FDA, have demonstrated that these GalNAc-conjugated, subcutaneously delivered siRNAs are well tolerated, significantly decrease the target mRNA levels, and have a low hazard profile. Other prominent companies have also utilized fully chemically modified, metabolically stabilized RNAi with varying secondary structures and chemical modification patterns. The RNAi therapy extends beyond the liver to other organs and targets various diseases ranging from rare to common diseases affecting larger patient populations. Quark Pharmaceuticals, for instance, has developed drugs to treat kidney injury (QPI-1002) and eye diseases (QPI-1007). Currently, the pharmaceutical industry’s focus has shifted swiftly toward RNAi drugs for cancer treatment. SiG12D LODER (Local Drug EluteR) is a biodegradable polymeric matrix that contains KRAS^G12D^ siRNA (siG12D) drug for the treatment of pancreatic ductal adenocarcinoma (NCT01188785) [136]. Additionally, TKM-080301(Plk1 inhibitor) has been developed for hepatocellular carcinoma and Atu027 (against protein kinase N3) for advanced solid tumors [137].

#### Beyond siRNA

miRNA inhibitors (Anti-miRs) and miRNA mimics can be used to down- or upregulate miRNAs. Miravirsen (SPC3649) and RG-101are anti-miRs targeting miR-122 for treating hepatitis C virus infection [138]. The miR-34a mimic, MRX34, is the first cancer-targeted miRNA drug [139]. However, none of them are currently in clinical usage

MTL-CEBPA is the first saRNA in clinical trials. It regulates hepatic and myeloid functions, as well as numerous oncogenic processes by upregulating the transcription factor CCAAT/enhancer-binding protein alpha (C/EBP-α) [140, 141]. The encouraging results promote the establishment of a clinical trial combining MTL-CEBPA with an anti-PD-1 checkpoint inhibitor or radiofrequency ablation to treat solid tumors [142].

### CRISPR/Cas-based genome therapy

The first human clinical trial on applying CRISPR/Cas gene editing involved using ex vivo Cas9 to knockout PD-1 in autologous T cells (NCT03399448) [143]. In addition, β-thalassemia was the first human trial applying CRISPR/Cas to genetic diseases (NCT03655678). The first clinical trial using CRISPR/Cas genome editing to treat retinal defects was EDIT-101 (NCT03872479) [144]. These CRISPR/Cas-based clinical trials have provided a foundation for further genome-editing clinical trials on zinc-finger nucleases [145].

### Aptamer

Pegaptanib (Macugen) is the first FDA-approval aptamer drug targeting VEGF to treat age-related macular degeneration [146]. Numerous other aptamers are in the preclinical or clinical development pipeline for possible treatment of diseases, such as visual disorders, coagulation, oncology and inflammation [54].

### mRNA vaccine

In the last few decades, mRNA vaccines have gained widespread applications. In 2020, the outbreak of coronavirus disease 2019 (COVID-19) stimulated the most rapid development of mRNA vaccines in history.

Vaccines targeting infectious diseases represent the most advanced application of mRNA therapies. So far, mRNA vaccines for various infectious diseases, including influenza, Zika, and respiratory syncytial virus, have been developed [57]. During the ongoing COVID-19 pandemic, mRNA-based vaccines have proven effective against the severe acute respiratory syndrome coronavirus 2 (SARS-CoV-2). The Pfizer–BioNTech vaccine BNT162b2 (Comirnaty^®^) [147] and the Moderna vaccine mRNA-1273 [148] were the first two FDA-approved SARS-CoV-2 vaccines with >94% effectiveness in phase III clinical trial. Both vaccines employ LNPs formulated using ionizable lipid and a nucleoside-modified mRNA. All uridines in the mRNA are substituted with N1-methylpseudouridine to improve mRNA translation. The mRNA sequence encodes the SARS-CoV-2 spike protein with two proline alterations that give the protein a prefusion shape [149].

The application of mRNA-based cancer vaccines has been recently reviewed [58, 73, 150, 151]. To date, over 20 mRNA-based vaccines have undergone clinical trials as potential preventive strategies for solid tumors, such as melanoma, non-small cell lung cancer, and colorectal carcinoma. In most clinical trials, mRNA cancer vaccines are co-administered with checkpoint modulators (PD-1, CTLA-4, and TIM3) or cytokine cocktails to enhance antitumor efficacy.

### More recent advances for RNA-based therapies

Apart from miRNA antagomir and miRNA mimic, artificial circular RNA (circRNA) sponges (circmiRs) are promising therapeutic miRNA antagonists. circRNAs are a subclass of ncRNAs that exist as continuous loop RNAs due to the lack of free 3′ and 5′ ends. They are resistant to nuclease degradation and more stable than linear RNAs. The most frequently described function of circRNAs is acting as miRNA sponges. These circRNAs contain multiple miRNA-binding sites for miRNAs binding, preventing the interaction between miRNA and their canonical mRNA target gene [152, 153]. Recent findings reveal that engineered circmiRs are efficient sponges of miR-132 and −212 to attenuate pressure overload-induced hypertrophy in vivo in a mouse model. These circmiRs also show greater in vitro efficacy than the current gold standard antagomiRs in inhibiting miRNA function [154]. Besides, circRNAs with minimized immunogenicity are potent protein kinase R inhibitors, which efficiently suppress protein kinase R activation 10^3^- to 10^6^-fold higher than reported chemical compounds (C16 and 2-AP) [155]. Moreover, circRNA vaccines against SARS-CoV-2 and emerging variants elicit a higher and more durable immune response [156]. In fact, increasing research has focused on noncoding RNA therapeutics, which have been widely reviewed [157–159].

Taken research advance of Cas13d mediated—genome therapy as another example. Cas13d has been shown to efficiently down-regulating cellular RNAs in mammalian cells in vitro [50]. The adeno-associated virus (AAV)-mediated delivery of CasRx and Pcsk9 sgRNAs into mouse liver has successfully decreased serum PCSK9 and serum cholesterol levels [160]. Besides, CasRx-mediated Vegfa knockdown in vivo could prevent the development of choroidal neovascularization in a mouse model of age-related macular degeneration [161]. The CasRx system-derived approach was more specific and efficient than RNAi-mediated gene knockdown. Therefore, CasRx-mediated therapy provides a robust method to silence genes in hepatocytes and other cells in vivo. Recently, it was proposed to be used for treating RNA virus infections (e.g., SARS-Cov-2).

## Conclusion and future perspectives

Various RNA-based approaches have been applied to experiments and clinical trials. Commoditized ASOs, siRNAs, antagomirs and aptamers are widely used for cell and animal experiments. Several ASOs, siRNAs, aptamer and mRNA vaccines have been approved for clinical application. These approaches allow for the down- or up-regulation of specific mRNA expression and inhibition of ncRNA functions by targeting particular RNA sequences. However, the most significant obstacle preventing the widespread usage of RNA-based approaches is the difficulty of efficiently delivering such drugs to target organs and tissues apart from the liver. In addition, off-target binding [162], sequence-induced toxicity, and oversaturation of the endogenous RNA processing pathway [163] affect the effectiveness of RNA-based approaches.

Chemical modification is one of the most promising strategies for delivering RNA-based drugs. Modification of the nucleic acid backbone, ribose ring, and nucleobase itself has been widely used to optimize drug-like characteristics for enhanced delivery. For example, extensive chemical modifications allow the delivery of gapmer ASOs to various tissues without an extra delivery agent. To date, eight of the ten approved oligonucleotide treatments are applied without an additional delivery vehicle. However, some unnatural nucleotides may be harmful. For instance, LNA-modified nucleic acids were found to cause severe hepatotoxicity [164]. Therefore, bioengineered RNAi agents (BERAs), a newly-developed in vivo RNA agent carrying no or minimal post-transcriptional modifications, have shown good application prospects [165].

Developing nanocarriers for RNA drug delivery provides excellent hepatic transport. Non-liver systemically administered nanomedicines require further investigation. Advances in imaging techniques (e.g., electron microscopy, super-resolution fluorescence microscopy, single-particle tracking 3, etc.) [166] and omics-based approaches [167] have allowed scientists to investigate intracellular delivery processes, thereby boosting the innovation of nanoformulations and rational design of advanced delivery vehicles.

Apart from the sequence-based method, small-molecule-based therapy to target RNA would be preferred in many cases [168]. Evrysdi (risdiplam) is the first FDA-approved orally bioavailable small-molecule inhibitor targeting *SMN2* pre-messenger RNA splicing. It is synthesized efficiently and can be easily administered [169]. Tutorials about generating RNA-targeted small molecules have been discussed in other reviews [5, 170].

In summary, combining RNA chemical modifications and conjugation with nanocarrier systems can improve the efficiency of RNA drug delivery. Further research on RNA-based therapeutics, including RNA molecules as therapeutic drugs and targeting RNA with small molecules, will lead to more RNA-based therapeutics for patient treatment.

## Data Availability

All relevant data are included in this paper.
